# Dual pathway inhibition as compared to acetylsalicylic acid monotherapy in relation to endothelial function in peripheral artery disease, a phase IV clinical trial

**DOI:** 10.3389/fcvm.2022.979819

**Published:** 2022-10-06

**Authors:** Loes H. Willems, Dick H. J. Thijssen, Laszlo A. Groh, Nina I. Kooijman, Hugo Ten Cate, Henri M. H. Spronk, A. Rogier T. Donders, Rozemarijn J. van der Vijver-Coppen, Frank van Hoek, Magdolna Nagy, Michel M. P. J. Reijnen, Michiel C. Warlé

**Affiliations:** ^1^Department of Surgery, Radboud University Medical Center, Nijmegen, Netherlands; ^2^Department of Physiology, Radboud Institute for Health Sciences, Radboud University Medical Center, Nijmegen, Netherlands; ^3^Research Institute for Sport and Exercise Sciences, Liverpool John Moores University, Liverpool, United Kingdom; ^4^Departments of Internal Medicine and Biochemistry, Maastricht University Medical Center (MUMC) and Cardiovascular Research Institute Maastricht (CARIM) School for Cardiovascular Diseases, Maastricht, Netherlands; ^5^Center for Thrombosis and Haemostasis, Gutenberg University Medical Center, Mainz, Germany; ^6^Department for Health Evidence, Radboud Institute for Health Sciences, Radboud University Medical Center, Nijmegen, Netherlands; ^7^Department of Surgery, Rijnstate Hospital, Arnhem, Netherlands; ^8^Multi-Modality Medical Imaging Group, Faculty of Science and Technology, University of Twente, Enschede, Netherlands

**Keywords:** peripheral arterial disease, aspirin, rivaroxaban, factor Xa inhibitors, endothelial cells, vascular endothelium

## Abstract

**Objective:**

Dual pathway inhibition (DPI) by combining acetylsalicylic acid (ASA) with low-dose rivaroxaban has been shown to reduce cardiovascular events in patients with peripheral arterial disease (PAD) when compared to ASA monotherapy. A potential explanation is that inhibition of factor Xa improves endothelial function through crosstalk between coagulation and inflammatory pathways, subsequently attenuating the occurrence of cardiovascular events. We hypothesize that the addition of rivaroxaban to ASA in PAD patients leads to improved endothelial function.

**Design:**

An investigator-initiated, multicentre trial investigating the effect of DPI on endothelial function.

**Methods:**

Patients, diagnosed with PAD, were enrolled in two cohorts: cohort A (Rutherford I-III) and cohort B (Rutherford IV-VI). Participants received ASA monotherapy for a 4-weeks run-in period, followed by 12 weeks of DPI. Macro- and microvascular endothelial dysfunction were studied by measuring carotid artery reactivity upon sympathetic stimulus and by measuring plasma endothelin-1 concentrations, respectively. All measurements were performed during the use of ASA (baseline) and after 12 weeks of DPI.

**Results:**

159 PAD patients (111 cohort A, 48 cohort B) were enrolled. Twenty patients discontinued study drugs early. Carotid artery constriction upon sympathetic stimulation at baseline (ASA) and after 12 weeks of DPI was similar in the total group, 22.0 vs. 22.7% (*p* = 1.000), and in the subgroups (Cohort A 22.6 vs. 23.7%, *p* = 1.000; cohort B 20.5 vs. 20.5%, *p* = 1.000), respectively. The mean concentration of plasma endothelin-1 at baseline and after 12 weeks of DPI did not differ, 1.70 ± 0.5 vs. 1.66 ± 0.64 pmol/L (*p* = *0.4*40) in the total group, 1.69 ± 0.59 vs. 1.62 ± 0.55 pmol/L in cohort A (*p* = 0.202), and 1.73 ± 0.53 vs. 1.77 ± 0.82 pmol/L in cohort B (*p* = *0.6*82), respectively.

**Conclusion:**

Macro- and microvascular endothelial dysfunction, as reflected by carotid artery reactivity and plasma endothelin-1 concentrations, are not influenced in PAD patients by addition of low-dose rivaroxaban to ASA monotherapy for 12 weeks.

**Trial registration:**

https://clinicaltrials.gov/ct2/show/NCT04218656.

## Introduction

Patients with peripheral arterial disease (PAD) are at high risk of developing cardiovascular events (1, 2), for which single antiplatelet therapy (SAPT) is indicated (3, 4). Recently, the COMPASS trial demonstrated that dual pathway inhibition (DPI), where acetylsalicylic acid (ASA) is combined with low-dose rivaroxaban (2.5 mg twice daily), reduces the rate of major adverse cardiovascular (MACE) and limb (MALE) events, compared to ASA monotherapy (5). The VOYAGER-PAD trial confirmed these findings in PAD patients, who underwent peripheral revascularization (6). The precise mechanism underlying the benefit of rivaroxaban in PAD patients is currently unclear.

Rivaroxaban is an oral inhibitor of factor Xa, the first enzyme in the “common pathway” of the coagulation cascade. Besides this prominent role in the coagulation cascade, previous literature demonstrates that factor Xa can activate protease-activated receptors (PAR) on the surface of endothelial cells in the inner lining of the arterial wall. By binding to PAR, factor Xa is capable of modulating inflammatory pathways, contributing to vascular inflammation, leukocyte migration and endothelial dysfunction (7–9).

Endothelial dysfunction contributes to the development and progression of atherosclerosis (10) and is generally present years before the patient develops symptomatic disease (11). Other risk factors for developing atherosclerotic disease have also been related to endothelial dysfunction, including hypertension (12), hyperlipidaemia (13), diabetes mellitus (14), and aging (15). Presence of endothelial dysfunction, independent from disease state and other risk factors, is strongly related to the occurrence of MACE (16–18) and MALE (16, 17). Risk-reducing interventions for atherosclerosis, such as physical activity and lipid-lowering drug therapy, have been shown to improve endothelial dysfunction (19–21). Rivaroxaban may therefore potentially improve endothelial function, thus contributing to the previously demonstrated reduction in MACE in PAD patients.

A simple and non-invasive test has been developed and validated to assess macrovascular endothelial function by measuring carotid artery reactivity (CAR) in response to a sympathetic stimulus. The CAR response was proven to be strongly related to cardiovascular risk, closely related to coronary artery endothelial function, and independently predictive for the 1-year occurrence of MACE in PAD patients (22, 23). Microvascular endothelial dysfunction, on the other hand, has been strongly related to plasma concentrations of endothelin-1 (ET-1) (24). ET-1 is a potent vasoconstrictor peptide (25) and has been suggested to be a potential target for treating microvascular endothelial dysfunction in atherosclerosis (26).

In this study, we hypothesize that DPI, by combining ASA with low-dose rivaroxaban for 12 weeks, reduces macro- and microvascular endothelial dysfunction in symptomatic PAD patients.

## Materials and methods

This is an investigator-initiated, non-randomized, multicenter parallel trial of two clinical cohorts investigating the effect of DPI (ASA with low-dose rivaroxaban) on endothelial function in PAD patients. The study was approved by the regional ethics committee and the local directory boards. The study was conducted in accordance with the latest revision of the Declaration of Helsinki and Good Clinical Practice regulations and is registered at ClinicalTrials.gov on January 6^th^, 2020 (NCT04218656) and at the Dutch Trial Register on September 22^nd^, 2020 (NL8908). Written informed consent was obtained from all participants. The protocol has been published previously (27).

### Participants

Patients with lower extremity PAD and an indication for SAPT according to the current guidelines (3, 4) were recruited. Based on the severity of PAD, patients were divided into cohort A (intermittent claudication, Rutherford I-III) and cohort B (chronic limb-threatening ischemia (CLTI), Rutherford IV-VI), where the highest Rutherford classification recorded for a patient (either now or in the past) was used to allocate a patient into one of the two cohorts (28). Patients with an increased bleeding risk, severe renal impairment, systemic treatment with CYP3A4 inhibitors/inducers, concomitant treatment with other anticoagulants, and known hypersensitivity to ASA/rivaroxaban were excluded. A detailed description of the in- and exclusion criteria can be found in our previously published protocol (27).

### Procedures

Eligible patients received low-dose (80–100 mg once daily) ASA monotherapy during a 4-weeks run-in period. After 4 weeks, medication adherence was evaluated by interview (T1). Participants with an adherence below 80% were excluded and replaced. Patients who dropped out before T1 and did not fulfill the run-in period were considered non-adherent and were replaced. Subsequently, at T1, participants were prescribed DPI by ASA (100 mg once daily) plus low-dose rivaroxaban (2.5 mg twice daily) for 12 weeks. After these 12 weeks (T2), DPI was discontinued, and participants could resume SAPT. During study participation, the occurrence of severe adverse events (SAE) was recorded, including myocardial infarction, stroke, acute limb ischemia (ALI), deterioration of PAD as classified by Rutherford (28), need for peripheral revascularization, lower extremity amputation, major bleeding and death.

### Outcomes

The primary outcome was a change in macrovascular endothelial dysfunction represented by a change in the proportion of participants with a constrictive CAR response upon sympathetic stimulus from T1 (ASA monotherapy) to T2 (after 12 weeks of DPI). The secondary outcome is a change in microvascular endothelial dysfunction represented by a change in mean plasma ET-1 level from T1 to T2.

We explored changes in the activation of the common pathway by measuring markers of thrombin generation, including thrombin:antithrombin (TAT) complexes and prothrombin fragment 1+2 (F1.2). Additionally, activated coagulation factor XI in complex with its natural inhibitor antithrombin (FXIa:AT) was measured, in order to assess for changes in activation of the intrinsic pathway (positive feedback loop mechanism).

### The carotid artery reactivity test

The CAR test assesses endothelial function by measuring the change in diameter of the common carotid artery in response to a sympathetic stimulus (cold pressor test). The common carotid artery was visualized using Philips Lumify ultrasound device (Philips Healthcare, Best, The Netherlands) with a L12-4 MHz linear array probe, during a 30 second baseline, and during a subsequent 3 mins of sympathetic stimulation by hand in ice water immersion. The common carotid artery diameter was measured with semi-automatic custom-designed edge-detection and wall-tracking software by an investigator, blinded for the test moment (T1 or T2). The area under the curve (AUC) relative to baseline was calculated. A net positive AUC represents a dilatory response, and a net negative AUC represents a constrictive response. Additionally, the peak change percentage dilatation or constriction (CAR%) was computed as the mean diameter over a 10-sec interval—excluding control interval—most deviating from baseline diameter, relative to baseline.

To limit the influence of external factors on the CAR test, participants were instructed 1) not to eat or drink anything except for water in the 6 h preceding their appointment, 2) not to have beverages with caffeine, alcohol, or any products that are high in vitamin C in the 18 h preceding their appointment, and 3) not to do heavy exercise training in the 24 h preceding their appointment. Each patient underwent endothelial testing twice, at T1 and at T2. Time of appointment was generally between 9 and 12 AM and was equal for both T1 and T2.

### Blood sampling

Venous blood samples were collected by venipuncture in 10 ml Lithium-Heparin (Vacuette) tubes. Platelet poor plasma was prepared by centrifuging whole blood at 2,500 g for 10 mins followed by a second centrifugation step at 2,500 g for 20 mins, both at room temperature. The platelet poor plasma was stored at −80°C until further analysis. Plasma concentrations of ET-1 were quantified using commercial ELISA kits (R&D, Minneapolis (Minnesota), United States of America). Plasma concentrations of TAT and FXIa:AT were quantified by in-house developed ELISA methods as described previously (29) and F1.2 was quantified using commercially available assays (Enzygnost™ F1+2, Siemens Healthineers, The Hague, the Netherlands).

### Statistical analyses

This study was powered to detect a relative reduction in the proportion of patients with CAR constriction when switching from ASA to DPI of ~40%. Based on previous literature, the expected prevalence of CAR constriction at baseline was 40% in cohort A (intermittent claudication) and 60% in cohort B (CLTI) (22). With an alpha of 5%, a power of 80%, and allowing for a drop-out rate of 5%, the final sample size was 159 participants (111 in cohort A and 48 in cohort B).

Change in proportion of participants with CAR constriction from T1 to T2 was compared using McNemar's Z-test, 2-sided equality. Differences in CAR% and plasma ET-1 levels from T1 to T2 was analyzed using the paired sample *t*-test. Differences in plasma concentrations of the coagulation markers TAT, F1.2 and FXIa:AT were explored using the Wilcoxon signed ranks test.

Categorical and continuous baseline variables are recorded as percentage and mean, respectively. SAEs were reported as numbers. Analyses are performed using IBM SPSS statistics 25. *p* values below 0.05 were considered significant.

## Results

### Enrolment and baseline characteristics

**Figure 1:**
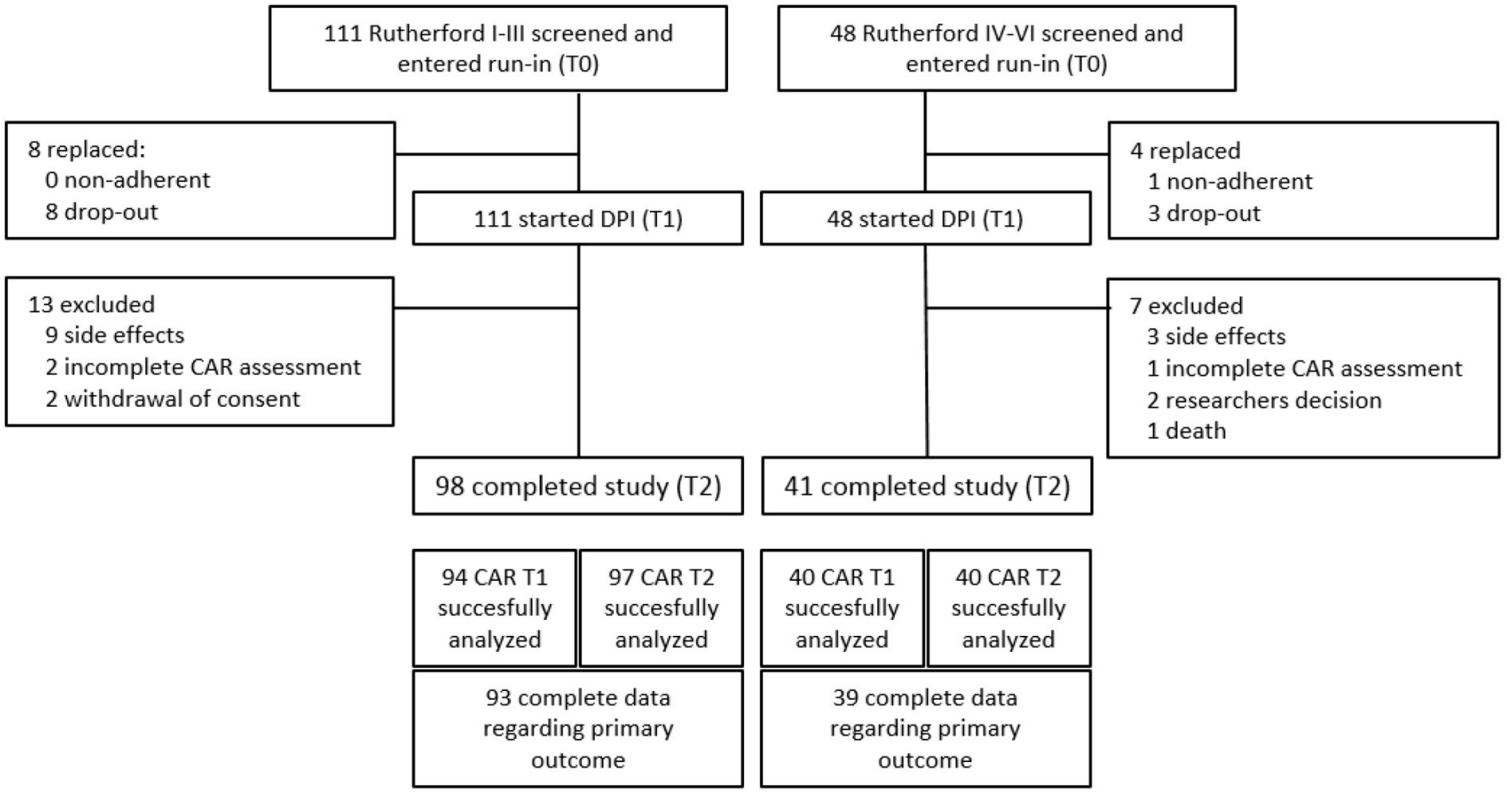
Flow diagram of enrolment and outcomes.

Between June 2020 and August 2021, 159 patients with symptomatic PAD were enrolled, with an additional 12 patients being enrolled to replace participants with an adherence below 80% at T1 (Figure 1). In total, 111 patients with intermittent claudication (cohort A) and 48 patients with CLTI (cohort B) completed the run-in period and were started on DPI. Of these, 20 discontinued the trial. The most common reasons for not continuing the trial were side effects associated with the study medication (Figure 1). Baseline characteristics are shown in Table 1. The mean age of the participants was 67 years and 66% were male. Most patients used ASA as SAPT (64.8%) before study participations, while 35.2% used clopidogrel.

**Table 1:** Baseline characteristics of the patients that successfully completed the run-in period.

	**All,** ***n* = 159**	**Cohort A,** ***n* = 111**	**Cohort B,** ***n* = 48**
Age (mean ± SD)	67 ± 8	67 ± 8	68 ± 9
Male (*n*, %)	105 (66.0)	77 (69.4)	28 (58.3)
BMI (mean ± SD)	26.8 ± 4.6	27.1 ± 4.7	26.1 ± 4.2
Tobacco use (*n*, %)			
Current	52 (32.7)	31 (27.9)	21 (43.8)
Former	98 (61.6)	74 (66.7)	24 (50.0)
Never	9 (5.7)	6 (5.4)	3 (6.3)
Alcohol use (*n*, %)			
Never	43 (27.0)	29 (26.1)	14 (29.2)
Rarely	17 (10.7)	11 (9.9)	6 (12.5)
Monthly	14 (8.8)	13 (11.7)	1 (2.1)
Weekly	46 (28.9)	33 (29.7)	13 (27.1)
Daily	39 (24.5)	25 (22.5)	14 (29.2)
Previous intervention for PAD (*n*, %)	105 (66.0)	64 (57.7)	41 (85.4)
Endovascular revascularization	100 (62.9)	64 (57.7)	36 (75.0)
Thrombendarteriectomy	12 (7.5)	(4.5)	7 (14.6)
Bypass surgery	18 (11.3)	6 (5.4)	12 (25.0)
Lower extremity amputation	5 (3.1)	0 (0.0)	5 (10.4)
Amputation of toe(s)	3 (1.9)	0 (0.0)	3 (6.3)
Thrombolysis	2 (1.3)	0 (0.0)	2 (4.2)
Embolectomy	1 (0.6)	0 (0.0)	1 (2.1)
Comorbidity (*n*, %)			
Hypertension	114 (71.7)	79 (71.2)	35 (72.9)
Hyperlipidaemia	71 (44.7)	51 (45.9)	20 (41.7)
Ischaemic heart disease	50 (31.4)	33 (29.7)	17 (35.4)
CVA/TIA	20 (12.6)	12 (10.8)	8 (16.7)
Diabetes mellitus	52 (32.7)	36 (32.4)	16 (33.3)
Asthma/COPD	42 (26.4)	30 (27.0)	12 (25.0)
Medication before study participation (*n*, %)			
Acetylsalicylic acid	103 (64.8)	75 (67.7)	28 (58.3)
Clopidogrel	56 (35.2)	36 (32.4)	20 (41.7)
Lipid lowering drugs	146 (91.8)	102 (91.9)	44 (91.7)
Antihypertensive drugs	117 (73.6)	82 (73.9)	35 (72.9)

Cohort A: intermittent claudication, Rutherford I-III. Cohort B: chronic limb-threatening ischaemia, Rutherford IV-VI.

BMI, body mass index; PAD, peripheral arterial disease; CVA, cerebrovascular accident; TIA, transient ischaemic attack; COPD, chronic obstructive pulmonary disease.

### Endothelial function

The CAR response was successfully assessed in 134/139 patients after a 4-weeks run-in period of ASA monotherapy (T1) and in 137/139 patients after 12 weeks of DPI (T2). Complete paired assessment was obtained of 132 participants, 93 in Cohort A and 39 in cohort B (Figure 1). The numbers of patients with a CAR dilatory response and a CAR constrictive response at T1 and T2, respectively, are presented in Table 2. The proportion of patients with a constrictive CAR response at T1 and T2 was similar in the total group, 22.0 vs. 22.7% (*p* = 1.000), respectively. Subsequent analysis on cohort A and cohort B revealed comparable trends in constrictive response rates, 22.6 vs. 23.7% (*p* = 1.000) for cohort A and 20.5 vs. 20.5% (*p* = 1.000) for cohort B, respectively. Consistent with this, the peak change in percentage of carotid artery diameter revealed no differences between T1 and T2 with a mean CAR% of 1.92 ± 2.88 vs. 1.69 ± 3.09 (*p* = 0.510) in the total group, 2.03 ± 2.98 vs. 1.71 ± 2.96 in cohort A (*p* = 0.442), and 1.66 ± 2.64 vs. 1.63 ± 3.42 in cohort B (*p* = 0.969), respectively (Table 3).

**Table 2:** Total patients with a CAR dilatory response and a constrictive response at the test moments T1 and T2.

**All participants**, ***n*** = **132**		**CAR response at T2, after 12 weeks of dual pathway inhibition**		
		**Dilatation**	**Constriction**	**Total**
CAR response at T1, after 4 weeks of ASA monotherapy	Dilatation	80	23	103
	Constriction	22	7	29
	Total	102	30	132
Cohort A, *n* = 93		CAR response at T2, after 12 weeks of dual pathway inhibition		
		Dilatation	Constriction	Total
CAR response at T1, after 4 weeks of ASA monotherapy	Dilatation	55	17	72
	Constriction	16	5	21
	Total	71	22	93
Cohort B, *n* = 39		CAR response at T2, after 12 weeks of dual pathway inhibition		
		Dilatation	Constriction	Total
CAR response at T1, after 4 weeks of ASA monotherapy	Dilatation	25	6	31
	Constriction	6	2	8
	Total	31	8	39

Cohort A: intermittent claudication, Rutherford I-III. Cohort B: chronic limb-threatening ischaemia, Rutherford IV-VI.

CAR, carotid artery reactivity; ASA, acetylsalicylic acid.

**Table 3:** The effect of DPI on endothelial dysfunction as represented by the CAR response, CAR%, and plasma endothelin-1 levels.

	**T1 (ASA)**	**T2 (DPI)**	***p***
CAR response, constriction, *n* (%)			*McNemar's test*
All	29 (22.0)	30 (22.7)	1.000
Cohort A	21 (22.6)	22 (23.7)	1.000
Cohort B	8 (20.5)	8 (20.5)	1.000
CAR%, mean ± SD			*Paired sample t-test*
All	1.92 ± 2.88	1.69 ± 3.09	0.510
Cohort A	2.03 ± 2.98	1.71 ± 2.96	0.442
Cohort B	1.66 ± 2.64	1.63 ± 3.42	0.969
Plasma endothelin-1, pg/mL, mean ± SD			*Paired sample t-test*
All	1.70 ± 0.57	1.66 ± 0.64	0.440
Cohort A	1.69 ± 0.59	1.62 ± 0.55	0.202
Cohort B	1.73 ± 0.53	1.77 ± 0.82	0.682

Cohort A: intermittent claudication, Rutherford I-III. Cohort B: chronic limb-threatening ischaemia, Rutherford IV-VI.

CAR, carotid artery reactivity; SD, standard deviation; ASA, acetylsalicylic acid; DPI, dual pathway inhibition.

The mean plasma ET-1 levels at T1 and T2 did not differ significantly and were 1.70 ± 0.57 vs. 1.66 ± 0.64 pg/mL (*p* = 0.440) for the total group, 1.69 ± 0.59 vs. 1.62 ± 0.55 pg/mL (*p* = *0.2*02) for cohort A, and 1.73 ± 0.53 vs. 1.77 ± 0.82 pg/mL (*p* = *0.6*82) for cohort B (Table 3).

### Coagulation activity

*In vivo* coagulation activity of the common and intrinsic pathway was measured at T1 and T2 (Table 4). The mean plasma concentration of TAT and F1.2 significantly decreased with DPI compared to ASA monotherapy, 1.13 ± 3.37 vs. 0.99 ± 3.82 μg/L (*p* = *0.0*13) and 386.43 ± 204.41 vs. 258.24 ± 153.79 pmol/L (p < *0.0*01), at T1 and T2 respectively. There was no significant difference in FXIa:AT levels between T1 and T2, 17.68 ± 25.69 vs. 16.90 ± 21.59 pM (*p* = *0.9*49).

**Table 4:** The effect of DPI on activation of the common and intrinsic pathway of coagulation.

	**T1 (ASA)**	**T2 (DPI)**	***p***
TAT, μg/L, mean ± SD	1.13 ± 3.37	0.99 ± 3.82	0.013
F1,2, pmol/L, mean ± SD	386.43 ± 204.41	258.24 ± 153.79	* <0.0*01
FXIa:AT, pM, mean ± SD	17.68 ± 25.69	16.90 ± 21.59	0.949

TAT, thrombin:antithrombin; F1,2, fragment 1+2; FXI:aAT, factorXI:antithrombin; SD, standard deviation; ASA, acetylsalicylic acid; DPI, dual pathway inhibition.

### Adverse events

In total, 18 SAEs were experienced by 16 patients (Supplementary Table S1). The two patients who experienced ALI had a second severe adverse event: one underwent a peripheral transluminal angioplasty of the iliac arteries, while the other decided for a palliative policy and died. Both patients used ASA before study participation and suffered from ALI during the run-in phase. The other 14 SAEs occurred during DPI treatment (Supplementary Table S1).

Possible side effects were reported by 31 participants, 4 of whom reported two possible side effects. The onset of the possible side effects was during DPI treatment for 28 of 31 participants. The most common side effects were minor bleeding problems (*n* = 11), skin rash (*n* = 6) and gastro-intestinal complaints (*n* = 5). Thirteen participants withdrew from study participation because of possible side effects of study medication (ASA 1, rivaroxaban 12).

## Discussion

In this study we investigated the potential impact of switching ASA monotherapy to DPI, by combining ASA with low-dose rivaroxaban, on macro- and microvascular endothelial dysfunction in patients with PAD and observed no differences. While the addition of rivaroxaban resulted in suppression of markers of thrombin formation, demonstrating an overall anticoagulant effect, the carotid artery response upon sympathetic stimulation and plasma ET-1 concentrations were similar after 4 weeks of ASA monotherapy and after 12 weeks of DPI.

Classically, macrovascular endothelial dysfunction was angiographically determined by detecting endothelium-dependent vasodilatation of the coronary arteries as response to an increase of endothelium derived nitric oxide by infusion of acetylcholine (30) or blood flow increasing medication (31). Due to risks related to the invasive character of these methods, non-invasive detection of endothelial dysfunction by vascular ultrasound gained interest. In this study, the CAR in response to cold pressor testing (sympathetic stimulus) was used. The CAR test is a simple, non-invasive test using an easy accessible vascular bed to assess macrovascular endothelial function. The CAR test closely relates to coronary artery endothelial function as tested by the classical invasive methods and has shown to be strongly related to cardiovascular risk in the population of peripheral arterial disease (22). Endothelial functions such as modulation of vascular tone, thrombogenicity and inflammation, are regulated by molecules, amongst which ET-1 (32). Plasma concentrations of ET-1 strongly relate to microvascular endothelial function (24) and are therefore an easy target to evaluate changes in its function. Previous research has even suggested ET-1 as a potential for treating microvascular endothelial dysfunction in atherosclerosis (26).

To our best knowledge, this study is the first to investigate changes in endothelial function by antithrombotic drugs. Previous literature has addressed the effect of lipid-lowering drugs on endothelial dysfunction. The impaired endothelial-dependent responses (i.e., nitric-oxide mediated vasodilatation), present in patients with hypercholesterolemia, can be reversed by lowering cholesterol levels using lipid-lowering therapies. This effect can already be observed after 1 month and persists with continued therapy (20, 33, 34). Furthermore, other cardiovascular risk reducing interventions have been shown to improve endothelial function. Exercise training augments endothelial dependent vasodilatation, provoked by acetylcholine infusion, in both coronary vessels and resistance vessels in atherosclerotic patients (19). The same effect can be observed by non-invasive methods of measuring endothelial dysfunction. Buckley et al. demonstrated significant improvements in vascular health after a 12-week physical activity program in patients with cardiovascular risk factors and in patients with manifest cardiovascular disease, with reversed carotid artery constriction in response to sympathetic stimulus and increased brachial artery flow-mediated dilatation (21, 35).

Improved endothelial dysfunction thus correlates with a reduction in cardiovascular risk factors and might subsequently reduce the occurrence of MACE. This is in line with the results of van Mil et al. who demonstrated that patients with a constrictive CAR response have a 4-fold increased risk of developing MACE and a 2-fold increased risk for clinical deterioration, in patients with manifest PAD. Low-dose rivaroxaban in addition to ASA has been shown to reduce the occurrence of myocardial infarction, stroke, ALI and cardiovascular death, in patients with PAD (5, 6). The current study, however, could not confirm an improvement in endothelial function underlying this benefit of rivaroxaban in PAD patients.

Rivaroxaban is an oral inhibitor of factor Xa, which plays a crucial role in the coagulation cascade by cleaving prothrombin, yielding the active thrombin. During this process, a fragment of prothrombin called F1,2 is released next to thrombin itself. Thrombin activates the intrinsic pathway through a positive feedback loop mechanism converting FXI into its active metabolite FXIa (36). Both thrombin and FXIa will be rapidly bound by antithrombin in circulation, generating TAT and FXI:AT, respectively. Treatment with rivaroxaban thus leads to a decrease in generation of active thrombin, coinciding with a decrease of TAT, FXIa, FXI:AT and F1,2. Since active thrombin is only present in circulation for a very short time, TAT, FXI:AT and F1,2 are acknowledge as more useful measures for thrombin level in the blood. A significant decrease in markers of thrombin generation is observed in participants at T2 compared to T1. This is consistent with the addition of rivaroxaban to ASA monotherapy and provides a strong indication that the negative findings regarding endothelial function in our study were not caused by non-adherence to medication. Since other studies on anticoagulants failed to show a clinical benefit on MACE in both the short- and long-term follow-up, it is highly unlike that the clinical benefit of rivaroxaban is fully explained by its effect on coagulation (37).

In addition to its role in the coagulation cascade, factor Xa has been identified as a direct agonist of PAR-1 leading to thrombin independent platelet activation and thrombus formation. By inhibiting factor Xa, rivaroxaban can attenuate platelet aggregation, in addition to its antithrombotic effect (7, 8, 38, 39). In mice, administration of rivaroxaban for 20 weeks reduced thrombus formation and atherosclerotic plaque destabilization (38, 40). In humans, platelet aggregation and thrombus formation under arterial flow conditions are attenuated in the presence of rivaroxaban, but thrombus regression has not been investigated (38).

Morphological improvement of atherosclerotic lesions has also been shown with dietary treatment in monkeys, and this improvement coincides with restoration of endothelial function (41). Since morphological improvement of atherosclerotic lesions has only been observed with long-term (20 weeks) treatment with rivaroxaban, the interventional period of 12-weeks DPI in the current study, might be too short to establish improved endothelial function. However, the clinical benefit of rivaroxaban as observed in the COMPASS and VOYAGER-PAD trial, is not delayed, but visible from study onset. Therefore, if improving endothelial dysfunction underlies the clinical benefit of rivaroxaban in PAD patients, one would expect to see some signs of improvement within a 12-week period of DPI.

Future research should address other possible long-term PAR-related benefits of rivaroxaban in humans, such as the capability of rivaroxaban to regress atherosclerotic plaques (42), and whether this relates to other (easily measurable) elements of PAR inhibition, such as improved endothelial function (after >12 weeks of rivaroxaban) or reduced vascular inflammation.

The strengths of this study are mainly related to its straightforward approach. By establishing a run-in period of ASA monotherapy, followed by 12 weeks of DPI, we could compare both antithrombotic strategies using paired assessments. Furthermore, the study protocol has been pre-published, facilitating replicability. There are also limitations that should be addressed. The prevalence of endothelial dysfunction, represented by CAR constriction, was lower than anticipated. While we predicted that 40% of the participants with claudication, and 60% of the participants with CLTI would show a constrictive response, in our study, the prevalence of CAR constriction varied between 20 and 25% in both cohorts. The expected high prevalence of CAR constriction was based on the CAVIPAD study, in which the CAR response was evaluated in 172 patients with PAD (22). A lower proportion of constrictive CAR in our study can be explained by our relatively strict in- and exclusion criteria. By selecting patients solely on SAPT, we implicitly may have excluded most patients with more severe (i.e., acute coronary syndrome in the past year, multivessel disease) concomitant coronary artery disease, and patients with a recent vascular intervention. Also, patients with a current malignancy and patients with a glomerular filtration rate below 30 were excluded. This might have led to the selection of a relatively “healthy” cohort of PAD patients. In addition, we classified patients into cohort A or B, based on their highest Rutherford classification ever, rather than on current classification. Therefore, patients in cohort B, might not have been as severely diseased as the patients with CLTI in the CAVIPAD study. A prevalence of 20–25% CAR constriction is in line with other studies that determined the CAR response in patients with atherosclerosis. The COVAS study, and the study by Buckley et al. both found a prevalence of 24% CAR constriction in respectively 50 patients with various expressions of atherosclerosis and 95 patients with coronary artery disease (35, 43). Another limitation is the relatively high drop-out rate. By replacing all participants that dropped out before starting DPI, we endeavored to obtain as much paired assessments of the primary and secondary outcomes as possible. Another 20 patients, however, dropped out during 12 weeks of DPI. Noteworthy, is the high number of side effects reported to the study team, which mainly underlies the high drop-out rate. Possible side effects of low-dose rivaroxaban were reported by 28 (17.6%) participants, and for 12 (7.5%) participants, these side effects were such that a stop in study medication was requested. The high occurrence of side effects should be considered when prescribing DPI in PAD patients. Last, some patients experienced peripheral revascularization during their DPI treatment. An eventual improvement of mobility with subsequent improvement in endothelial function, was not corrected for. As the number of patients undergoing revascularization was relatively small we believe that this was not a relevant source of bias, although a certain influence cannot be ruled out

In conclusion, macro- and microvascular endothelial dysfunction, as determined by determining the CAR response and measuring plasma ET-1 concentrations, is not influenced by addition of low-dose rivaroxaban to ASA monotherapy during 12 weeks in PAD patients.

## Data availability statement

The raw data supporting the conclusions of this article will be made available by the authors, without undue reservation.

## Ethics statement

The studies involving human participants were reviewed and approved by Medical Research Ethics Committee Oost-Nederland. The patients/participants provided their written informed consent to participate in this study.

## Author contributions

LW, DT, ARD, MR, and MW contributed to the concept of the study and the study design. LW, NK, RV-C, FH, MR, and MW contributed to the data collection. LW, LG, ARD, and MN contributed to the data analyses. LW, DT, LG, HT, HS, MN, MR, and MW contributed to the data interpretation. LW wrote the first version of the manuscript. All authors read, revised, and approved the final manuscript.

## Funding

This work was supported by Bayer B.V. (Grant Number 21065). The funder was not involved in the study design, collection, analysis, interpretation of data, the writing of this article or the decision to submit it for publication.

## Conflict of interest

The authors declare that the research was conducted in the absence of any commercial or financial relationships that could be construed as a potential conflict of interest.

## Publisher's note

All claims expressed in this article are solely those of the authors and do not necessarily represent those of their affiliated organizations, or those of the publisher, the editors and the reviewers. Any product that may be evaluated in this article, or claim that may be made by its manufacturer, is not guaranteed or endorsed by the publisher.
