# Supplementary material for: Dual pathway inhibition as compared to acetylsalicylic acid monotherapy in relation to endothelial function in peripheral artery disease, a phase IV clinical trial
Source: Front Cardiovasc Med. 2022 Oct 6;9:979819. doi: 10.3389/fcvm.2022.979819 (PMC9583941; doi:10.3389/fcvm.2022.979819)
Supplement: Supplementary file 1 [file Table_1.docx]

**Supplementary table 1** severe adverse events and possible side effects of study drugs.

| Severe adverse events | Total number | Onset during run-in | Onset during DPI |
| --- | --- | --- | --- |
| Acute limb ischemia  Peripheral revascularization  Cardiovascular death  Non-cardiovascular death  Gastro-intestinal haemorrhage | 2  13  1  1  1 | 2  1  1 | 12  1  1 |
| Possible side effects | Total number | Onset during ASA | Onset during DPI |
| Bleeding problems  Dysmenorrhoea  Anaemia  Bleeding haemorrhoids  Gastro-intestinal haemorrhage  Easy bruising  Haematuria  Epistaxis  Skin rash  Gastro-intestinal  Stomach complaints  Obstipation  Palpitations  Arm, leg and/or back pain  Dizziness  Headache  Shortness of breath  Decreased kidney function  Fatigue | 11  2  1  1  1  3  1  2  6  5  4  1  1  5  2  2  1  1  1 | 1  1  1 | 11  2  1  1  1  3  1  2  5  5  3  1  1  5  2  2  1  1 |
